# Estimating the Characteristic Curve of a Directional Control Valve in a Combined Multibody and Hydraulic System Using an Augmented Discrete Extended Kalman Filter

**DOI:** 10.3390/s21155029

**Published:** 2021-07-24

**Authors:** Qasim Khadim, Mehran Kiani-Oshtorjani, Suraj Jaiswal, Marko K. Matikainen, Aki Mikkola

**Affiliations:** 1 Department of Mechanical Engineering, LUT School of Energy Systems, Lappeenranta University of Technology, 53850 Lappeenranta, Finland; qasim.khadim@lut.fi (Q.K.); Suraj.Jaiswal@lut.fi (S.J.); marko.matikainen@lut.fi (M.K.M.); aki.mikkola@lut.fi (A.M.); 2 Department of Energy Technology, LUT School of Energy Systems, Lappeenranta University of Technology, 53850 Lappeenranta, Finland

**Keywords:** parameter estimation, curve fitting method, multibody dynamics, hydraulic system, predictive maintenance, characteristic curve, product life cycle, digital twin

## Abstract

The estimation of the parameters of a simulation model such that the model’s behaviour matches closely with reality can be a cumbersome task. This is due to the fact that a number of model parameters cannot be directly measured, and such parameters might change during the course of operation in a real system. Friction between different machine components is one example of these parameters. This can be due to a number of reasons, such as wear. Nevertheless, if one is able to accurately define all necessary parameters, essential information about the performance of the system machinery can be acquired. This information can be, in turn, utilised for product-specific tuning or predictive maintenance. To estimate parameters, the augmented discrete extended Kalman filter with a curve fitting method can be used, as demonstrated in this paper. In this study, the proposed estimation algorithm is applied to estimate the characteristic curves of a directional control valve in a four-bar mechanism actuated by a fluid power system. The mechanism is modelled by using the double-step semi-recursive multibody formulation, whereas the fluid power system under study is modelled by employing the lumped fluid theory. In practise, the characteristic curves of a directional control valve is described by three to six data control points of a third-order B-spline curve in the augmented discrete extended Kalman filter. The results demonstrate that the highly non-linear unknown characteristic curves can be estimated by using the proposed parameter estimation algorithm. It is also demonstrated that the root mean square error associated with the estimation of the characteristic curve is 0.08% with respect to the real model. In addition, all the errors in the estimated states and parameters of the system are within the 95% confidence interval. The estimation of the characteristic curve in a hydraulic valve can provide essential information for performance monitoring and maintenance applications.

## 1. Introduction

Multibody system dynamics (MBS) approaches enable the creation of the equations of motion that describe a mechanical system and relevant sub-components of complex mechanical systems [1,2]. The use of MBS leads to physics-based models that act as a single source of information [3] and represent the operation of an equivalent physical system in the real world [4]. The data generated by an MBS simulation model can be used to solve real-world problems throughout a product’s life cycle [5].

A physical system might have parameters that are difficult to estimate and that could accordingly create uncertainties in MBS models. In the real world, these parameters might be cumbersome or sometimes even impossible to measure directly due to economical limitations and sensor implementation difficulties. In addition, these parameters might change over time due to wear and other factors that come into play during operation. In some cases, parameters can only be interpreted from the manufacturer’s catalogues while not manifesting the current state of a product or differences in individual products due to manufacturing tolerances. Estimating these parameters can provide valuable information about the state and working performance of a product [6,7]. Manufacturers can use this information for condition monitoring [8,9], predictive maintenance [10,11,12], and real-time simulations for digital-twin applications [13,14].

In general, parameter estimation is a discipline that provides the essential tools for the estimation of parameters appearing in the modelling of a system [15]. The most common techniques for parameter estimation are weighted least squares [16,17], Kalman filtering [18,19], orthogonal least squares [20], robust techniques that include clustering [10], and regression diagnostics [21]. Among these algorithms, Kalman filters for parameter estimation have been utilised in a wide variety of engineering studies, ranging from control [22] and mechatronics [23] to heat transfer [24,25], fluid mechanics [26,27], turbulence [28], and others.

In the MBS field, several types of Kalman filter algorithms have been used to estimate system states based on the multibody equations of motion [29,30,31,32,33,34]. In state estimation, the independent coordinate method was introduced by using the independent positions and velocities of the multibody model as the states of the Kalman filter [30]. Using the independent coordinate method, the MBS formulation offers a general approach for estimating the system coordinates in terms of independent states for open- and closed-loop systems [30,31,34]. Less attention has been given to parameter estimation in MBS systems [35]. This is due to the complexity of the problem. As in many cases, the parameters are not constant and have to be estimated from the measurable variables of the dynamic system. In an-MBS related study, vehicle suspended mass and road friction were estimated in a dual-estimation application by using the extended Kalman filter (EKF) and the unscented Kalman filter (UKF) [36]. The generalised polynomial chaos (gPC) theory was first implemented in the framework of MBS in 2006 to quantify the parametric and external uncertainties [37,38]. However, in [37,38], only constant parameters were estimated.

Contrary to this, in practical systems, the parameters are a function of several system variables and may follow very complicated and unknown non-linear variations during the working cycles [39,40]. For instance, in the case of a hydraulically actuated mobile working machine, the characteristic curve of a hydraulic valve can play a significant role in terms of machine performance [41,42]. The characteristic curve of a hydraulic valve can be expressed as a function of the spool position and the semi-empiric flow rate coefficient [41,42]. The semi-empiric flow rate coefficient relates the discharge coefficient, pressure losses, and flow characteristics that demonstrate the dynamic characteristics of a hydraulic valve [43,44,45]. Accordingly, the characteristic curve of a hydraulic valve can be used in the condition monitoring and predictive maintenance of hydraulically driven systems [42]. However, in an operating hydraulic system, only the minimum and maximum points on the characteristic curve can be determined from the manufacturer’s catalogues with a high level of certainty [41,42]. The characteristic curve of a hydraulic valve remains unclear in a working cycle and varies from one hydraulic valve to another due to manufacturing tolerances and possible wear [41,42]. Applying parameter estimation theories [46,47] in combination with MBS equations of motion can enable the estimation of the characteristic curve of a hydraulically driven physical system in operation by using a limited amount of information.

Generally, unknown parameters are treated as constants in the dynamic equations of motion. The estimation of non-linear parameters typically requires an accurate description of the first derivatives of the corresponding parameters. However, in the real world, the first derivatives of parameters are unclear. The first derivative of a characteristic curve in a hydraulic valve is an example. In the case of a characteristic curve, a vector of data points can be constructed using random points between the minimum and maximum values provided in the manufacturer’s catalogues. Through a parameter vector, the characteristic curve of a hydraulic valve in the real world can be estimated by combining parameter estimation algorithms [46,47] and curve-fitting methods [48,49,50,51,52]. Considering parameter estimation constraints, this study proposes the estimation of parameters by combining the augmented discrete extended Kalman filter (ADEKF) with curve-fitting methods.

The objective of this study is to propose a parameter estimation algorithm by combining the ADEKF algorithm with a curve-fitting method in an application for estimating linear and non-linear parameters. To this end, parameters are introduced as vectors in the augmented state vector. Due to the accuracy of the finite difference schemes in the complex plane, as demonstrated in [53,54], an approach to computing the Jacobian of a non-linear system of ordinary differential equations (ODEs) through complex variables in the framework of a parameter estimation algorithm is proposed. Based on the parameter estimation algorithm, the structures of covariance matrices of plant and measurement noises are introduced. The parameter estimation algorithm is applied to estimate the characteristic curve of a directional control valve in a hydraulically driven four-bar mechanism. As reported in [55], the double-step formulation has advantages over Index-3 Augmented Lagrangian formulation due to the use of a coordinate partitioning method [56]. Therefore, the double-step semi-recursive formulation is used to model the four-bar mechanism with relative coordinates. A fluid power system, in turn, is modelled by using the lumped fluid theory. This algorithm is verified by estimating the characteristic curves of the directional control valve using three, four, five, and six vector data control points in the mechanism. The implementation of the parameter estimation algorithm is explained by using MBS simulation models that represent the real model, estimation model, and simulation model. The estimation model considers the actuator position, pump pressure, and the pressure on the piston side as sensor measurements to account for the system responses. Applying the proposed parameter estimation methodology in MBS systems can enable the estimation of parameters of any complex system in a real-world system.

The rest of this paper is organised as follows. In Section 2, the parameter estimation methodology is described. Section 2 details further into the double-step semi-recursive MBS formulation, lumped fluid theory, monolithic approach, the ADEKF with a curve-fitting method, and structure of covariance matrices of plant and measurement noises. The parameter estimation methodology is applied to the case example presented in Section 3. Section 4 demonstrates the results of the parameter estimation algorithm for the case example. Finally, conclusions about parameter estimation are provided in Section 5.

## 2. Parameter Estimation Methodology

**Figure 1:**
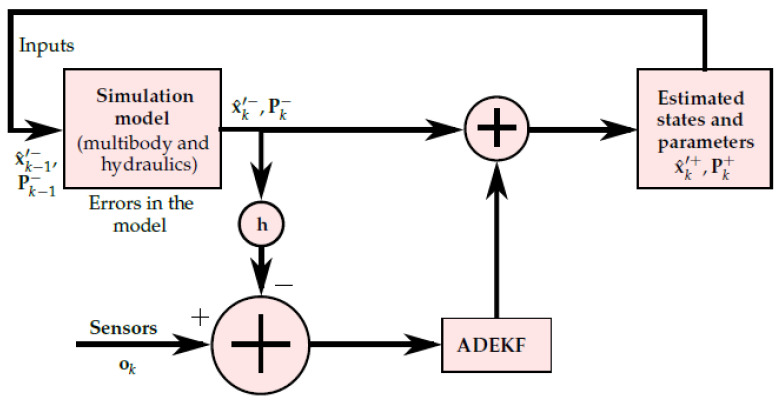
Parameter estimation methodology.

Figure 1 depicts a methodology that can be used to estimate the parameters of a dynamic system by using a simulation model. In this model, an initial covariance matrix $P_{k-1}^{+}\in R^{L\times L}$ and an augmented state vector ${\hat{x}}_{k-1}^{\prime +}={\left[\begin{array}{cc} x_{k-1}^{T} & y_{k-1}^{T} \end{array}\right]}^{T}$ at the time step k−1 are introduced. Here, *L* is the dimension of the augmented state vector, and R denotes the set of real numbers. $x\in R^{L-n_{h_{p}}}$ and $y\in R^{n_{h_{p}}}$ represent the states and parameters of the system, respectively. Here, $n_{h_{p}}$ is the number of hydraulic parameters.

In the real world, the sensors shown in the Figure 1 can be replaced by sensor measurements obtained from a physical system, such as a forklift, a tractor, etc. [4,57]. To account for the system response, the sensor measurement vector o includes the minimum number of measurements required by the ADEKF algorithm to estimate the states and parameters of a real system. In Figure 1, h corresponds to the sensor measurement function. Note that the parameters should not be included in the measurement vector, i.e., y∉o. The parameter estimation algorithm estimates the augmented state vector ${\hat{x}}_{k}^{\prime +}$ and covariance matrix $P_{k}^{+}$ from the minimum information of the real system at time step k−1 in the simulation model.

### 2.1. Multibody Dynamic Formulations

The parameter estimation methodology described in Section 2 is applied to the simulation of a hydraulically driven mechanism. In this study, the hydraulically driven mechanism is modelled using a double-step semi-recursive MBS formulation and the lumped fluid theory. The coupled multibody and hydraulic dynamics are integrated by using a single-step implicit trapezoidal integration scheme in a monolithic coupling approach. As demonstrated in [55], the double-step semi-recursive formulation uses a coordinate partitioning method [58,59,60] to express the hydraulically driven mechanism in terms of independent coordinates. As a result, the double-step semi-recursive formulation presents an appropriate multibody simulation approach for state and parameter estimation applications.

#### 2.1.1. Double-Step Semi-Recursive Formulation

In the semi-recursive formulation, a body *i* is defined by the set of six Cartesian velocities as $Z_{i}={\left[\begin{array}{cc} {\dot{r}}_{i}^{T} & \omega_{i}^{T} \end{array}\right]}^{T}$ and six Cartesian accelerations as ${\dot{Z}}_{i}={\left[\begin{array}{cc} {\ddot{r}}_{i}^{T} & {\dot{\omega }}_{i}^{T} \end{array}\right]}^{T}$ for a complete description [61,62]. Here, ${\dot{r}}_{i}$, ${\ddot{r}}_{i}$, $\omega_{i}$ and ${\dot{\omega }}_{i}$ are velocities, accelerations, angular velocities, and angular accelerations of the body, respectively. In the relative coordinate system, the position of $n_{b}$ bodies in a system can be described by using joint coordinates as $z={\left[\begin{array}{cccc} z_{1} & z_{2} & \ldots & z_{n_{b}} \end{array}\right]}^{T}$ [61,62]. The absolute velocity Z and the absolute acceleration $\dot{Z}$ of the system bodies can be mapped in terms of the relative joint velocity vector $\dot{z}$ and the relative joint acceleration vector $\ddot{z}$ by using the velocity transformation matrix as follows [61,62]:

(1) $$\left.\begin{array}{cc} Z\; & =TR_{d}\dot{z}\\ \dot{Z}\; & =TR_{d}\ddot{z}+T{\dot{R}}_{d}\dot{z} \end{array}\;\right\},$$

where $T\in R^{6n_{b}\times 6n_{b}}$ is the path matrix that demonstrates the topology of the system, and $R_{d}\in R^{6n_{b}\times n_{b}}$ is a block diagonal matrix. The path matrix T is a lower triangular matrix and contains entries of 6×6 $\left(I_{6}\right)$ unit matrices representing bodies between the body under observation and the root of the system [61]. In Equation (1), the block diagonal matrix $R_{d}$ and the product ${\dot{R}}_{d}\dot{z}$ can be computed with the joint-dependent element of the velocity transformation matrix $b_{i}\in R^{6\times 1}$ and the joint-dependent element of the acceleration transformation vector $d_{i}\in R^{6\times 1}$, respectively [61,62]. The semi-recursive formulation is described hereafter, but the interested reader is referred to [61,62] for further details of T, $b_{i}$, and $d_{i}$. The composite mass matrix ${\bar{M}}_{i}\in R^{6\times 6}$ and the composite force vector ${\bar{Q}}_{i}\in R^{6\times 1}$ of the *i*th body can be described using absolute coordinates as [61,62]:

(2) $${\bar{M}}_{i}=\left[\begin{array}{cc} m_{i}I_{3} & -m_{i}{\tilde{g}}_{i}\\ m_{i}{\tilde{g}}_{i} & J_{i}-m_{i}{\tilde{g}}_{i}{\tilde{g}}_{i} \end{array}\right],$$

(3) $${\bar{Q}}_{i}=\left[\begin{array}{c} {\bar{f}}_{i}-{\tilde{\omega }}_{i}\left({\tilde{\omega }}_{i}m_{i}g_{i}\right)\\ n_{i}-{\tilde{\omega }}_{i}J_{i}\omega_{i}+{\tilde{g}}_{i}\left(f_{i}-{\tilde{\omega }}_{i}\left({\tilde{\omega }}_{i}m_{i}g_{i}\right)\right) \end{array}\right],$$

where $m_{i}$ is the mass of the *i* body, ${\bar{f}}_{i}\in R^{3\times 1}$ is a vector of external forces, ${\tilde{\omega }}_{i}$ is the skew-symmetric matrix of the angular velocity vector, $n_{i}\in R^{3\times 1}$ is the vector of external moments, $I_{3}$ is a 3×3 unit matrix, and ${\tilde{g}}_{i}\in R^{3\times 3}$ is the skew-symmetric matrix of the position vector of the centre of mass of the body in the global coordinate system. In Equation (2), $J_{i}$ is the inertia tensor of the *i*th body, which can be computed as described in [61]. Applying the principle of virtual work and using Equation (1) yields the equation of motion for an open-loop system in the simplified form [61,62]:

(4) $${R_{d}}^{T}T^{T}\bar{M}TR_{d}\ddot{z}={R_{d}}^{T}T^{T}\left(\bar{Q}-\bar{M}T{\dot{R}}_{d}\dot{z}\right),$$

where $\bar{M}\in R^{6n_{b}\times 6n_{b}}$ is the block diagonal matrix consisting of the composite mass matrices of the bodies. The force vector $\bar{Q}\in R^{6n_{b}\times 1}$ is the column matrix of composite forces. To incorporate closed-loop systems, the double-step semi-recursive formulation is used in this study [62]. In this method, a set of *m* constraint equations $\Phi \left(z\right)\;=\;0$ are introduced for closure of an open-loop system. This method employs Gaussian elimination with a full pivoting approach to identify the independent and dependent columns of the Jacobian matrix $\Phi_{z}$ [62,63,64]. Through this formulation, relative joint-independent coordinates can be used to define the dynamics of a system, i.e., the relative joint-dependent coordinates can be computed in terms of the relative joint-independent coordinates. Hence, this provides an appropriate option for the state and parameter estimation applications. The relative joint velocity vector $\dot{z}$ can be described using the coordinate partitioning method [59]:

(5) $$\left[\begin{array}{c} {\dot{z}}^{d}\\ {\dot{z}}^{i} \end{array}\right]=\left[\begin{array}{c} -{\left(\Phi_{z}^{d}\right)}^{-1}\Phi_{z}^{i}\\ I \end{array}\right]{\dot{z}}^{i}\equiv R_{z}{\dot{z}}^{i},$$

where ${\dot{z}}^{d}\in R^{m}$ are the relative joint-dependent velocities, ${\dot{z}}^{i}\in R^{n_{f}}$ are the relative joint-independent velocities, $R_{z}\in R^{n_{b}\times n_{f}}$ is the velocity transformation matrix, $\Phi_{z}^{d}\in R^{m\times m}$, and $\Phi_{z}^{i}\in R^{m\times n_{f}}$ are the Jacobian matrices of the constraint equations with respect to the dependent and independent relative joint positions, respectively. In Equation (5), it is assumed that neither singular configurations nor redundant constraints exist; as a consequence, the inverse of matrix $\Phi_{z}^{d}$ can be obtained [55,59]. The relative joint acceleration vector can be expressed by differentiating Equation (5) [59]:

(6) $$\ddot{z}=R_{z}{\ddot{z}}^{i}+{\dot{R}}_{z}{\dot{z}}^{i},$$

where ${\ddot{z}}^{i}$ are the relative joint-independent accelerations, and ${\dot{R}}_{z}$ is the derivative of the velocity transformation matrix. Substituting Equation (6) into Equation (4) results in an equation of motion for a closed-loop system in a simplified form [55,56,58,63,64]:

(7) $$R_{z}^{T}R_{d}^{T}T^{T}\bar{M}TR_{d}R_{z}{\ddot{z}}^{i}=R_{z}^{T}R_{d}^{T}\left(T^{T}\bar{Q}-T^{T}\bar{M}D\right),$$

where $D=TR_{d}\left[\begin{array}{c} -{\left(\Phi_{z}^{d}\right)}^{-1}\left({\dot{\Phi }}_{z}\dot{z}\right)\\ 0 \end{array}\right]+T{\dot{R}}_{d}\dot{z}$ represent the absolute accelerations when the vector $\ddot{z}$ is zero in Equation (6). Equation (7) can be further simplified using the accumulated mass matrix $M^{\Sigma }=R_{z}^{T}R_{d}^{T}T^{T}\bar{M}TR_{d}R_{z}$ and the accumulated force matrix $Q^{\Sigma }=R_{z}^{T}R_{d}^{T}\left(T^{T}\bar{Q}-T^{T}\bar{M}D\right)$.

#### 2.1.2. Hydraulic Lumped Fluid Theory

The lumped fluid theory can be used to compute pressures within a hydraulic circuit [65]. Using this approach, a hydraulic circuit is assumed to be composed of discrete volumes. The pressures inside the volumes are equally distributed, with the acoustic waves having a negligible effect on the pressures [41,65]. In any hydraulic volume $V_{h}$, the differential pressure ${\dot{p}}_{h}$ can be computed [41,65] as

(8) $${\dot{p}}_{h}=\frac{k_{p}+p_{h}k_{0}}{V_{h}}\sum_{h=1}^{n_{f}}Q_{h},$$

where $Q_{h}$ is the sum of incoming and outgoing volume flow rates, $k_{0}$ is the flow gain, $k_{p}$ is the pressure flow coefficient, and $n_{f}$ is the total amount of volume flows. By employing a semi-empirical method, the volume flow rate $Q_{R}$ through a throttle valve can be described as [66]

(9) $$Q_{R}=C_{R}\;\mathrm{sgn}(\Delta p)\;\sqrt{|\Delta p|},$$

where Δp is the pressure difference and $C_{R}=C_{d}A_{R}\sqrt{\frac{2}{\rho }}$ is the semi-empirical flow rate coefficient of the throttle valve. Here, $C_{d}$ is the flow discharge coefficient and ρ is the fluid density. In Equation (9), $A_{R}$ is the cross-sectional area of the pressure relief valve. Similarly, the volume flow rate $Q_{d}$ through a directional control valve can be computed as [67,68]

(10) $$Q_{d}=C_{v}U\;\mathrm{sgn}(\Delta p)\;\sqrt{|\Delta p|},$$

where $C_{v}$ is the semi-empiric flow rate coefficient, and *U* is the relative position of the spool. Equation (10) is complemented by the following first-order differential equation:

(11) $$\dot{U}=\frac{U_{ref}-U}{\tau },$$

where $U_{ref}$ is the reference voltage signal, and τ is the time constant. The incoming flow rate $Q_{in}$ and outgoing flow rate $Q_{out}$ in the hydraulic cylinder can be described as

(12) $$Q_{in}=\dot{s}A_{1},\;Q_{out}=\dot{s}A_{2},$$

where $\dot{s}$ is the piston velocity, and $A_{1}$ and $A_{2}$ are the areas on the piston and piston-rod side of the cylinder, respectively. The force $F_{h}$ produced by the cylinder can be written as

(13) $$F_{h}=p_{1}A_{1}-p_{2}A_{2}-F_{\mu },$$

where $p_{1}$ and $p_{2}$ are, respectively, the pressure on the piston and piston-rod side, which can be calculated by using Equation (8). $F_{\mu }$ is the total friction force in the hydraulic cylinder caused by the hydraulic sealing. As proposed in [69], this friction force can be calculated by employing the Brown and McPhee model [70], which is valid for both positive and negative tangential velocity. The actuator velocity dependent friction force can be written in the vector form as

(14) $$F_{\mu }=\left(F_{c}\tanh\left(4\frac{\left\|\dot{s}\right\|}{v_{s}}\right)+\left(F_{s}-F_{c}\right)\frac{\frac{\left\|\dot{s}\right\|}{v_{s}}}{{\left(\begin{array}{c} \frac{1}{4}{\left(\frac{\left\|\dot{s}\right\|}{v_{s}}\right)}^{2}+\frac{3}{4} \end{array}\right)}^{2}}\right)\mathrm{sgn}\left(\dot{s}\right)+\sigma_{2}\dot{s}\tanh\left(4\right),$$

where $F_{c}$ is the Coulomb friction, $v_{s}$ is the Stribeck velocity, $F_{s}$ is the static friction, $\sigma_{2}$ is the coefficient of viscous friction, and $\dot{s}$ is the actuator velocity vector.

#### 2.1.3. Monolithic Approach: Coupling MBS and Hydraulic Dynamic Systems

The MBS formulation can be combined with the fluid power system solver to form a unified set of non-linear differential equations in a monolithic approach:

(15) $$\left.\begin{array}{cc} M^{\Sigma }\left(z\right){\ddot{z}}^{i} & =Q^{\Sigma }\left(z,\dot{z},p\right)\\ \dot{p} & =v(z,\dot{z},p,y,U) \end{array}\;\right\},$$

where p is the pressure vector, and y is the vector of hydraulic parameters. Equation (15) is a set of non-linear equations that can be represented as $f(\bar{x},U)=0$. Here, the vector $\bar{x}={\left[\begin{array}{cccc} z^{T} & {\dot{z}}^{T} & p^{T} & y^{T} \end{array}\right]}^{T}$. The solution of the non-linear equations described in Equation (15) is stiff. A stiff equation can be solved by using single-step implicit trapezoidal integration scheme [55,71,72,73]. In this integration scheme, the relative joint-independent positions and the pressures are initially predicted as $z_{k+1}^{i}=z_{k}^{i}+{\dot{z}}_{k}^{i}\Delta k+\frac{1}{2}{\ddot{z}}_{k}^{i}\Delta k^{2}$ and $p_{k+1}=p_{k}+{\dot{p}}_{k}\Delta k$, respectively [73]. The derivatives of $z_{k+1}^{i}$ and $p_{k+1}$ can be predicted as

(16) $$\left.\begin{array}{cc} {\dot{z}}_{k+1}^{i} & =\frac{2}{\Delta k}z_{k+1}^{i}+{\overset{ˇ}{\dot{z}}}_{k}^{i}\\ {\ddot{z}}_{k+1}^{i} & =\frac{4}{\Delta k^{2}}z_{k+1}^{i}+{\overset{ˇ}{\ddot{z}}}_{k}^{i}\\ {\dot{p}}_{k+1} & =\frac{2}{\Delta k}p_{k+1}+{\overset{ˇ}{\dot{p}}}_{k} \end{array}\;\right\},$$

where ${\overset{ˇ}{\dot{z}}}_{k}^{i}=-\left(\frac{2}{\Delta k}z_{k}^{i}+{\dot{z}}_{k}^{i}\right)$, ${\overset{ˇ}{\ddot{z}}}_{k}^{i}=-\left(\frac{4}{\Delta k^{2}}z_{k}^{i}+\frac{4}{\Delta k}{\dot{z}}_{k}^{i}+{\ddot{z}}_{k}^{i}\right)$ and ${\overset{ˇ}{\dot{p}}}_{k}=-\left(\frac{2}{\Delta k}p_{k}+{\dot{p}}_{k}\right)$. Note that the relative joint-dependent positions $z_{k+1}^{d}$ are obtained from $z_{k+1}^{i}$ and the previous step $z_{k}^{d}$ by solving the position problem $\Phi \left(z\right)\;=\;0$ [59,60,61]. The non-linear constraint equations are solved iteratively with the Newton–Raphson method [59,60,61]. The derivatives of the relative joint-dependent positions $z_{k+1}^{d}$ are computed from Equations (5) and (6) at the velocity and acceleration levels, respectively [55]. Substituting Equation (16) into Equation (15) leads to a set of dynamic equilibrium equations as $\bar{F}\left(\chi_{k+1}\right)=0$ at the time step k+1 as

(17) $$\left.\begin{array}{cc} M^{\Sigma }z_{k+1}^{i}-\frac{\Delta k^{2}}{4}Q_{k+1}^{\Sigma }+\frac{\Delta k^{2}}{4}M^{\Sigma }{\overset{ˇ}{\ddot{z}}}_{k}^{i} & =0\\ \frac{\Delta k}{2}p_{k+1}-\frac{\Delta k^{2}}{4}v_{k+1}+\frac{\Delta k^{2}}{4}{\overset{ˇ}{\dot{p}}}_{k} & =0 \end{array}\;\right\},$$

where $\chi_{k+1}={\left[\begin{array}{cc} {\left(z^{i}\right)}_{k+1}^{T} & p_{k+1}^{T} \end{array}\right]}^{T}$ is unknown. The Newton–Raphson method is employed on the non-linear Equation (17) to iteratively compute the unknown variables [73,74]:

(18) $${\left[\begin{array}{c} \frac{\partial \bar{F}\left(\chi \right)}{\partial \chi } \end{array}\right]}_{k+1}^{\left(h\right)}\Delta \chi_{k+1}^{\left(h\right)}=-{\left[\begin{array}{c} \bar{F}\left(\chi \right) \end{array}\right]}_{k+1}^{\left(h\right)},$$

where $\left\|\Delta z_{k+1}^{i}\right\|<1\times 10^{-10}\;\mathrm{rad}$ and $\left\|\Delta p_{k+1}\right\|<1\times 10^{-2}\;\mathrm{Pa}$ are the error tolerances in the relative joint independent positions and pressures provided in the Newton–Raphson method. In Equation (18), ${\left[\begin{array}{c} \bar{F}\left(\chi \right) \end{array}\right]}_{k+1}^{\left(h\right)}$ is the residual vector, which can be computed as

(19) $${\left[\begin{array}{c} \bar{F}\left(\chi \right) \end{array}\right]}_{k+1}^{\left(h\right)}=\frac{\Delta k^{2}}{4}{\left[\begin{array}{c} M^{\Sigma }{\ddot{z}}^{i}-Q^{\Sigma }\\ \dot{p}-v \end{array}\right]}_{k+1}^{\left(h\right)}.$$

In Equation (18), ${\left[\begin{array}{c} \frac{\partial \bar{F}\left(\chi \right)}{\partial \chi } \end{array}\right]}_{k+1}^{\left(h\right)}$ is the tangent matrix, which can be numerically approximated at a point $\chi_{0}$ by using a forward finite differences, as demonstrated in the literature [72,75]. Now, by computing $\Delta \chi_{k+1}^{\left(h+1\right)}$ from Equation (18), the next iteration $\chi_{k+1}^{\left(h+1\right)}$ can be calculated.

### 2.2. Estimation Algorithm: ADEKF with a Curve-Fitting Method

In this section, the ADEKF parameter estimation algorithm is introduced in the framework of a B-spline curve-fitting method. It is important to note, however, that the introduced procedure can be easily modified for applications of other curve-fitting methods, as mentioned in [48,49]. Parameter estimation through the ADEKF comprises prediction and update stages. At the prediction stage, in the case of the coupled multibody and hydraulic systems, the augmented state vector can be described as $x^{\prime }={\left[\begin{array}{cccc} {\left(z^{i}\right)}^{T} & {\left({\dot{z}}^{i}\right)}^{T} & p^{T} & y^{T} \end{array}\right]}^{T}$. At this step, ${\hat{x}}_{k}^{\prime -}$ is calculated in the time integration of a dynamic model described as [46]

(20) $${\hat{x}}_{k}^{\prime -}=f\left({\hat{x}}_{k-1}^{\prime +},U_{k}\right),$$

To account for unknown parameters, the proposed parameter estimation algorithm employs the curve-fitting method. Through this method, a B-spline curve is constructed with the knot vector u for non-uniform open splines [48,49] at the current time step:

(21) $$C\left(u\right)=\sum_{i=0}^{n}B^{i,d}\left(u\right)N^{i},$$

where n is the number of control points, *d* is the degree, $B^{i,d}\left(u\right)$ are the *d*th order of B-spline basis functions, and $N^{i}$ is the control point vector. The control point vector can be expressed in terms of the system parameters y. For instance, in the case of the characteristic curve, the control point vector can be written in terms of the spool position and semi-empiric flow rate coefficient as $N=\left[\begin{array}{ccccc} U_{\min} & U_{1} & \ldots & U_{n} & U_{\max}\\ C_{v_{\min}} & C_{v_{1}} & \ldots & C_{v_{n}} & C_{v_{\max}} \end{array}\right]$. Here, $U_{\min},U_{1}$, and $U_{n}$ represent spool positions, and $C_{v_{\min}},C_{v_{1}}$, and $C_{v_{\max}}$ are the semi-empiric flow rate coefficients of a hydraulic valve. $B^{i,d}\left(u\right)$ can be defined by using the Cox–de Boor recursion formula [48,49]:

(22) $$B^{i,0}\left(u\right)=\left\{\begin{array}{cc} 1 & u^{i}\leq u<u^{i+1}\\ 0, & \mathrm{otherwise} \end{array}\right.,$$

(23) $$B^{i,j}\left(u\right)=\frac{u-u^{i}}{u^{i+j}-u^{i}}B^{i,j-1}\left(u\right)+\frac{u^{i+j+1}-u}{u^{i+j+1}-u^{i+1}}B^{i+1,j-1}\left(u\right),$$

where $u^{i}$ is the *i*th element of the knot vector for non-uniform open splines. Next, the numerical values of parameters, which are scalar, should be evaluated by using Equation (21) at time step *k* to be incorporated in Equation (20). The calculation of Equation (20) at the desired input signal is straightforward. However, the numerical computation of the Jacobian matrix $f_{x_{k-1}^{\prime }}$ could be challenging when using a curve-fitting method. Each term of the Jacobian matrix can be approximated by using complex variables to reduce the truncation error [53,54] for very small increments. For instance, in the case of a multi-variable function, the Jacobian column of Equation (20) with respect to the *r*th term of the augmented state vector ${{\hat{x}}^{\prime }}_{k-1}$ can be written in the partial derivative form as

(24) $$\frac{\partial f({\hat{x}}_{k-1,1}^{\prime },{\hat{x}}_{k-1,2}^{\prime },\ldots ,{\hat{x}}_{k-1,L}^{\prime })}{\partial {\hat{x}}_{k-1,r}^{\prime }}=\frac{Im(f\left({\hat{x}}_{k-1,1}^{\prime +},{\hat{x}}_{k-1,2}^{\prime +},\ldots ,{\hat{x}}_{k-1,r}^{\prime }+i\delta ,\ldots ,{\hat{x}}_{k-1,L}^{\prime +}\right))}{\delta }+O\left(\delta^{2}\right),$$

where $r\in \left[1,L\right]$, and iδ represents a very small increment in the complex plane. δ=Lε is a real value. Epsilon ε is a very small number and depends on the machine’s precision. The *r*th term of the state vector ${\hat{x}}_{k-1,r}^{\prime }+i\delta$ is expanded by using the Taylor series [53,54].

The evaluation of $Im(f\left({\hat{x}}_{k-1,1}^{\prime +},{\hat{x}}_{k-1,2}^{\prime +},\ldots ,{\hat{x}}_{k-1,r}^{\prime }+i\delta ,\ldots ,{\hat{x}}_{k-1,L}^{\prime +}\right))$ for the parameter vector $u_{k}$ requires the evaluation of $C(u_{k-1})$ as complex numbers. However, the knot vector cannot contain any complex numbers [48,49]. Therefore, a criterion $|t-t_{k-1}^{i}|<\Xi$ is introduced, where Ξ can be a small real number, such as 0.1, which implies that the knot-point distance between $t_{k-1}^{i}$ and the complex input argument *t* should be less than Ξ. Using this criterion enables the knot points to be evaluated with the curve-fitting method through complex input. With the Jacobian of the dynamic system $f_{x_{k-1}^{\prime }}$, the covariance matrix $P_{k}^{-}$ is approximated as [46]

(25) $$P_{k}^{-}=f_{x_{k-1}^{\prime }}P_{k-1}^{+}f_{x_{k-1}^{\prime }}^{T}+Q_{k},$$

where $Q_{k}$ is the covariance matrix of the plant noise. In the update stage, the sensor measurements are taken into account to improve the estimated augmented state vector ${\hat{x}}_{k}^{\prime -}$. The innovation $\Delta_{k}$ is calculated as [46]

(26) $$\Delta_{k}=o_{k}-h\left({\hat{x}}_{k}^{\prime -}\right),$$

where $o_{k}$ are the sensor measurements at the *k* time step, and $h(x_{k}^{\prime -})$ is the sensor measurement function. With the Jacobian of the sensor measurement function $h_{x^{\prime }}$, the innovation in the covariance matrix $S_{k}$ and the Kalman gain $K_{k}$ can be calculated as [46]

(27) $$\left.\begin{array}{cc} S_{k} & =h_{x_{k}^{\prime }}P_{k}^{-}h_{x_{k}^{\prime }}^{T}+R_{k}\\ K_{k} & =P_{k}^{-}h_{x_{k}^{\prime }}^{T}S_{k}^{-1} \end{array}\;\right\},$$

where $R_{k}$ is the covariance matrix of the measurement noise. Finally, the augmented state vector ${\hat{x}}_{k}^{\prime +}$ and covariance matrix $P_{k}^{+}$ are updated at the time step *k*, which will be used for the next time step as [46]

(28) $$\left.\begin{array}{cc} {\hat{x}}_{k}^{\prime +} & ={\hat{x}}_{k}^{\prime -}+K_{k}\Delta_{k}\\ P_{k}^{+} & =\left(I_{L}-K_{k}h_{x_{k}^{\prime }}\right)P_{k}^{-} \end{array}\;\right\},$$

where $I_{L}$ is the identity matrix of dimension *L*.

#### Covariance Matrices of Process and Measurement Noises

It is well known that when applying Kalman filters, the tuning of the filter parameters is crucial, especially the covariance matrices of the plant and measurement noises. Furthermore, it was established in [30,31] that in dealing with non-linear systems, the improper tuning/setting of these covariance matrices can make the algorithm unstable. In this study, the properties of measurement noise are precisely known because the measurements are built from a dynamic model (providing ground truth) with an addition of white Gaussian noise to replicate real sensors. Thus, the covariance matrix of measurement noise can be obtained. For instance, when using position and pressure sensors, the covariance matrix of the measurement noise, R, would then take the form [30,31,34]

(29) $$R=\left[\begin{array}{cc} {\left(\begin{array}{c} \sigma_{s}^{\prime } \end{array}\right)}^{2}I_{n} & 0_{n\times n_{p}}\\ 0_{n_{p}\times n} & {\left(\begin{array}{c} \sigma_{p}^{\prime } \end{array}\right)}^{2}I_{n_{p}} \end{array}\right],$$

where $\sigma_{s}^{\prime }$ and $\sigma_{p}^{\prime }$ are the standard deviations of measurement noises at the position and pressure levels, respectively. In Equation (29), *n* is the number of actuator sensors and $n_{p}$ is the number of pressure sensors. $I_{n}$, $I_{n_{p}}$, $0_{n\times n_{p}}$, and $0_{n_{p}\times n}$ are the identity and zero matrices of corresponding orders, respectively. In the case of a multibody model along with positions and velocities as the state vector, the structure of the plant noise in the discrete-time frame was well established in [30,31] and can be written as

(30) $$Q=\left[\begin{array}{ccc} \frac{\sigma_{\ddot{x}}^{2}\Delta t^{3}I_{n_{f}}}{3} & \frac{\sigma_{\ddot{x}}^{2}\Delta t^{2}I_{n_{f}}}{2}\\ \frac{\sigma_{\ddot{x}}^{2}\Delta t^{2}I_{n_{f}}}{2} & \sigma_{\ddot{x}}^{2}\Delta tI_{n_{f}} & \end{array}\right],$$

where Δt is the size of the integration time step and $n_{f}$ is the number of degrees of freedom of the system. It should be noted that Equation (30) includes the variance at the acceleration level, $\sigma_{\ddot{x}}$, because of the acceleration errors arising from the inaccurate description of forces and mass distribution. Furthermore, the state vector in this study also includes the hydraulic pressures and the hydraulic parameters, along with the positions and velocities, and errors can occur at the pressure and parameter levels as well. Therefore, inspired by [34], the variance of hydraulic pressures, $\sigma_{p,D}$, and the variance of hydraulic parameters, $\sigma_{h_{p,D}}$, can be directly incorporated as the diagonal elements in Equation (30). Accordingly, the structure of the covariance matrix of the plant noise, Q, in the parameter estimation can be written as

(31) $$Q=\left[\begin{array}{cccc} \frac{\sigma_{\ddot{x}}^{2}\Delta t^{3}I_{n_{f}}}{3} & \frac{\sigma_{\ddot{x}}^{2}\Delta t^{2}I_{n_{f}}}{2} & 0_{n_{f}\times n_{p}+n_{h_{p}}} & 0_{n_{f}\times n_{p}+n_{h_{p}}}\\ \frac{\sigma_{\ddot{x}}^{2}\Delta t^{2}I_{n_{f}}}{2} & \sigma_{\ddot{x}}^{2}\Delta tI_{n_{f}} & 0_{n_{f}\times n_{p}+n_{h_{p}}} & 0_{n_{f}\times n_{p}+n_{h_{p}}}\\ 0_{n_{p}+n_{h_{p}}\times n_{f}} & 0_{n_{p}+n_{h_{p}}\times n_{f}} & \sigma_{p,D}^{2} & 0_{n_{f}\times n_{p}+n_{h_{p}}}\\ 0_{n_{p}+n_{h_{p}}\times n_{f}} & 0_{n_{p}+n_{h_{p}}\times n_{f}} & 0_{n_{p}+n_{h_{p}}\times n_{f}} & \sigma_{h_{p,D}}^{2} \end{array}\right].$$

In this study, the integration errors are assumed to be negligible in comparison to the acceleration, pressure, and parameter errors.

## 3. Case Example: Hydraulically Actuated System

**Figure 2:**
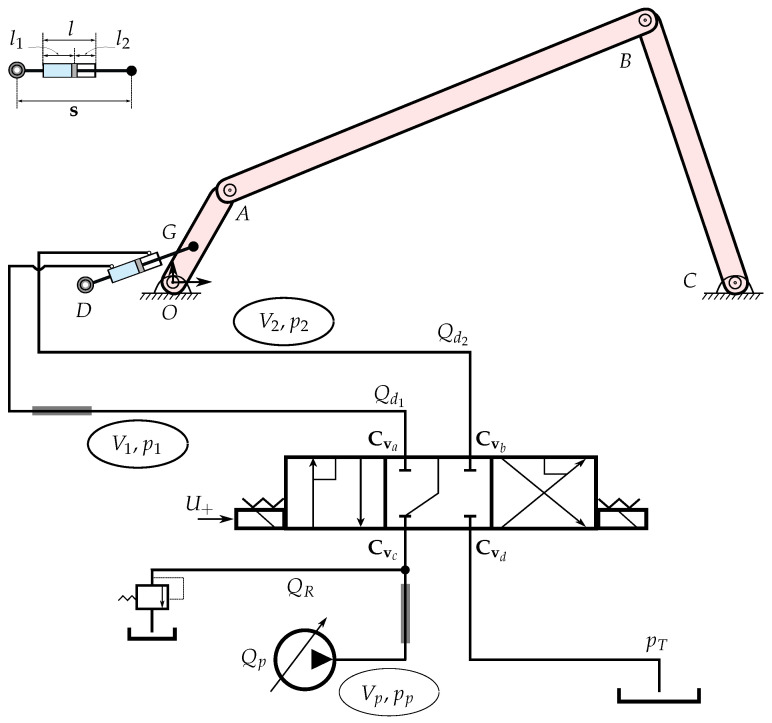
Hydraulically actuated four-bar mechanism. The mechanism is actuated by a differential pressure pump. $C_{v_{a}},C_{v_{b}},C_{v_{c}},$ and $C_{v_{d}}$ represent the semi-empiric flow rate coefficients at the *a*, *b*, *c*, and *d* ports of the 4/3 directional control valve. Grey rectangles indicate the pressure sensors on the control volumes $V_{p}$ and $V_{1}$.

The parameter estimation methodology described in Section 2 is applied to estimate the characteristic curves at the *a*, *b*, *c*, and *d* ports of the 4/3 directional control valve shown in Figure 2. A four-bar mechanism actuated by a hydraulic circuit is presented in Figure 2. The dynamics of the mechanism are modelled using the semi-recursive and hydraulic lumped fluid theories, as described below.

### 3.1. Dynamic Model of the System

The bodies of the mechanism are assumed to be rectangular beams whose lengths are $L_{1}=2\;m$, $L_{2}=8\;m$, and $L_{3}=5\;m$ and whose masses are $m_{1}=100\;kg$, $m_{2}=400\;kg$, and $m_{3}=250\;kg$, respectively. The position vector at point *D* is $r_{D}={\left[\begin{array}{ccc} -\frac{L_{1}}{2} & 0 & 0 \end{array}\right]}^{T}$. The point *G* is located at the centre of mass of body 1. The double-step semi-recursive formulation described in Section 2.1.1 is used to model the four-bar mechanism.

The four-bar mechanism is actuated using the sinusoidal reference input signal, which is taken as $U_{ref}=10\sin\left(0.4\pi k\right)$, where *k* is the simulation run time. The simulations are performed for 5 s. The hydraulic circuit consists of a double-acting hydraulic cylinder, connecting hoses 1 and 2, a 4/3 directional control valve, a pressure relief valve, a connecting hose of volume $V_{p}$, a differential pump of pressure $p_{p}$, and a tank with a constant pressure source $p_{T}$.

The lumped fluid theory described in Section 2.1.2 is used to compute the pressures within the hydraulic circuit. In the application of the lumped fluid theory, the hydraulic circuit can be divided into three control volumes $V_{p}$, $V_{1}$, and $V_{2}$. The pressure derivatives ${\dot{p}}_{p}$, ${\dot{p}}_{1}$, and ${\dot{p}}_{2}$ through these volumes can be computed as

(32) $$\left.\begin{array}{cc} {\dot{p}}_{p} & =\frac{k_{p}+p_{p}k_{0}}{V_{p}}\left(Q_{p}-Q_{R}-Q_{d_{1}}\right)\\ {\dot{p}}_{1} & =\frac{k_{p}+p_{1}k_{0}}{V_{1}}\left(Q_{d_{1}}-A_{1}\dot{s}\right)\\ {\dot{p}}_{2} & =\frac{k_{p}+p_{2}k_{0}}{V_{2}}\left(A_{2}\dot{s}-Q_{d_{2}}\right) \end{array}\;\right\},$$

where $Q_{d_{1}}$ and $Q_{d_{2}}$ are the flow rates in the control volumes 1 and 2. In Equation (32), $Q_{p}$ and $Q_{R}$ are the pump flow rate and flow rate through the pressure relief valve, respectively. The flow rates $Q_{R}$, $Q_{d_{1}}$, and $Q_{d_{2}}$ can be computed by employing Equations (9) and (10), respectively. The constant hydraulic parameters are tabulated in Table 1. In Equation (32), $\dot{s}$ is the actuator velocity, which can be determined from the actuator position vector s. Following Figure 2, the vector s can be calculated from the position vectors $r_{G}$ and $r_{D}$ as

(33) $$\left.\begin{array}{cc} s & =r_{G}-r_{D}\\ \dot{s} & =\frac{d|s|}{\Delta t}=\dot{s}\;\cdot \;\frac{s}{|s|}={\dot{r}}_{G}\;\cdot \;\frac{s}{|s|} \end{array}\;\right\},$$

where ${\dot{r}}_{G}$ is the velocity vector of point *G*. The control volumes $V_{1}$ and $V_{2}$ appearing in Equation (32) can be calculated as follows:

(34) $$\left.\begin{array}{cc} V_{1} & =V_{h_{1}}+A_{1}l_{1}\\ V_{2} & =V_{h_{2}}+A_{2}l_{2} \end{array}\;\right\},$$

where $V_{h_{1}}$, $V_{h_{2}}$, and $V_{p}$ are the control volumes of the respective hoses, as described in Table 1. In Equation (34), $l_{1}$ and $l_{2}$ are the lengths of the piston side and the piston-rod side chambers, respectively. $l_{1}$ and $l_{2}$ can be calculated with the vector s as

(35) $$\left.\begin{array}{cc} l_{1} & =l_{1_{0}}-\left|s\right|+s_{0}\\ l_{2} & =l_{2_{0}}+\left|s\right|-s_{0} \end{array}\;\right\},$$

where $l_{1_{0}}$ and $l_{2_{0}}$ are the initial piston side length and the initial piston-rod side length, respectively. $l_{1_{0}}$ and $l_{2_{0}}$ are computed from the length of cylinder *l*, which is given in Table 1.

Using the vector s, the hydraulic force $F_{h}$ produced by the double-acting cylinder can be calculated as

(36) $$F_{h}={\left[\begin{array}{ccc} \frac{s_{X}}{|s|}F_{h} & \frac{s_{Y}}{|s|}F_{h} & \frac{s_{Z}}{|h|}F_{h} \end{array}\right]}^{T},$$

where $F_{h}$ is computed from Equation (13). The hydraulic force vector $F_{h}$ is combined with the external force vector $f_{i}$ to calculate $\bar{Q}$ in Equation (3). The resultant equations of motion (15) are formulated for the hydraulically driven four-bar mechanism. Equations (15) are solved by using an implicit single-step trapezoidal integration scheme in a monolithic approach, which was described in Section 2.1.3.

#### 3.1.1. Real and Estimation Models

In this study, three dynamic versions of the mechanism are used to demonstrate the implementation of the parameter estimation algorithm. One of the models is the real model. The sensor measurements o are taken from the real model. The modelling errors are introduced in the force model of the estimation model with respect to the real model. The properties of the estimation model and the simulation model are the same. In Table 2, the properties of the real model, the estimation model, and the simulation model are provided. Note that the simulation model is used in this study to demonstrate the differences between the simulated world and the real world.

As in practise, the minimum and maximum points on the characteristic curves of a directional control valve can be determined from the manufacturer’s catalogues. Using this limited information, the characteristic curves are defined linearly at all ports of the directional control valve in the cases of the estimation model and the simulation model. The linear characteristic curves are implemented by using the minimum and maximum values of the semi-empiric flow rate coefficients $C_{v_{a}}$, $C_{v_{b}}$, $C_{v_{c}}$, and $C_{v_{d}}$ at the valve closing and the valve opening positions, respectively. The linear characteristic curves of the directional control valve affect the dynamics of the estimation model throughout the simulation runtime. In the case of the real model, the characteristic curves of the directional control valve are unclear and can be non-linear. With Equation (21), the non-linear characteristic curves of the directional control valve are implemented using $C_{v_{a}}$, $C_{v_{b}}$, $C_{v_{c}}$, and $C_{v_{d}}$ in the hydraulic circuit of the real model. Similarly, the initial actuator positions $s_{1_{0}}$ of the real model and the estimation model are different. Note that the initial relative joint coordinates of the bodies in the system can be found from $s_{1_{0}}$ and ${\dot{s}}_{1_{0}}$ by using geometrical relationships. To avoid instabilities in the integration process, the simulations are started in the static equilibrium position, the details of the mechanism of which can be found in [34].

#### 3.1.2. Sensor Measurements

In this study, the measurable observations $o={\left[\begin{array}{ccc} s & p_{p} & p_{1} \end{array}\right]}^{T}$ are taken from the real model. In the real model, the actuator position sensor measures the actuator position *s* [76]. Gauge pressure sensors are used for the pressure measurements $p_{p}$ and $p_{1}$ [34]. These pressure sensors measure the pressure with respect to the atmospheric pressure. The pressure sensors are installed on their respective volumes, as also shown in Figure 2. The numerical values of the standard deviation, as mentioned in Equation (29), are taken as $\sigma_{s}^{\prime }=1.12\times 10^{-3}\;m$ and $\sigma_{p}^{\prime }=1.5\times 10^{5}$ Pa for the actuator and pressure sensors, respectively.

### 3.2. Parameter Estimation Algorithm

In the parameter estimation algorithm, the augmented state vector ${\hat{x}}^{\prime }$ is defined as

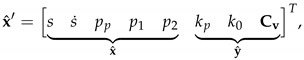
(37)
where *s* is the actuator position, $\dot{s}$ is the actuator velocity, $p_{p}$, $p_{1}$, and $p_{2}$ are the pressures, $k_{p}$ is the pressure flow coefficient, $k_{0}$ is the flow gain, and $C_{v}=\left[\begin{array}{cccc} C_{v_{a}} & C_{v_{b}} & C_{v_{c}} & C_{v_{d}} \end{array}\right]$ are the semi-empiric flow rate coefficients at the corresponding ports of the directional control valve. In Equation (37), $\hat{x}$ and $\hat{y}$ present the states and the parameters of the hydraulically driven four-bar mechanism, respectively. Equations (20)–(28) are implemented to estimate the augmented state vector ${\hat{x}}^{\prime }$ and the characteristic curves of the directional control valve. In this application, the third-order B-spline interpolation method is combined with the ADEKF. For the case example, three, four, five, and six control points are used in the parameter estimation algorithm to compute Equations (20) and (24). As mentioned earlier, the first, third, and fourth state variables are measured. Therefore, the sensor measurement function $h({\hat{x}}_{k}^{\prime -})$ and its Jacobian $h_{x^{\prime }}$ can be written as

(38) $$\left.\begin{array}{cc} h({\hat{x}}_{k}^{\prime -}) & ={\left[\begin{array}{ccc} {\hat{x}}_{k,1}^{\prime -} & {\hat{x}}_{k,3}^{\prime -} & {\hat{x}}_{k,4}^{\prime -} \end{array}\right]}^{T}\\ h_{x^{\prime }} & =\left[\begin{array}{ccccccccccc} 1 & 0 & 0 & 0 & 0 & 0 & 0 & \frac{\partial h}{\partial C_{v_{a}}} & \frac{\partial h}{\partial C_{v_{b}}} & \frac{\partial h}{\partial C_{v_{c}}} & \frac{\partial h}{\partial C_{v_{d}}}\\ 0 & 0 & 1 & 0 & 0 & 0 & 0 & \frac{\partial h}{\partial C_{v_{a}}} & \frac{\partial h}{\partial C_{v_{b}}} & \frac{\partial h}{\partial C_{v_{c}}} & \frac{\partial h}{\partial C_{v_{d}}}\\ 0 & 0 & 0 & 1 & 0 & 0 & 0 & \frac{\partial h}{\partial C_{v_{a}}} & \frac{\partial h}{\partial C_{v_{b}}} & \frac{\partial h}{\partial C_{v_{c}}} & \frac{\partial h}{\partial C_{v_{d}}} \end{array}\right] \end{array}\;\right\},$$

where $C_{v}=\left[\begin{array}{cccc} C_{v_{a}} & C_{v_{b}} & C_{v_{c}} & C_{v_{d}} \end{array}\right]$. $h({\hat{x}}_{k}^{\prime -})$ and $h_{x^{\prime }}$ are used in Equations (26) and (27), respectively, in the parameter estimation algorithm.

## 4. Results and Discussion

In this section, the results of the simulation of the estimation model of the parameter estimation algorithm are presented. The results of the estimation model are compared to those of the real model and the simulation model. The initial covariance $P_{0}$ used in the augmented state estimator includes $\sigma_{s}^{2}=1\times 10^{-4}\;m^{2}$ for the actuator position, $\sigma_{\dot{s}}^{2}=1\times 10^{-4}\;m^{2}/s^{2}$ for the actuator velocity, and three pressure terms of $\sigma_{p}^{2}=22.50\times 10^{7}\;{\mathrm{Pa}}^{2}$ in the diagonal. For the hydraulic parameters, the initial covariance values $\sigma_{k_{p}}^{2}=1\times 10^{14}\;{\mathrm{Pa}}^{2}$, $\sigma_{k_{0}}^{2}=1\times 10^{2}$ and $\sigma_{C_{v}}^{2}=9\times 10^{2}\;\frac{m^{6}}{s^{2}Pa}$ are used in the diagonal. The numerical values of the plant noise $\sigma_{\ddot{x}}^{2}=0.8\;m^{2}/s^{4}$ and $\sigma_{p}^{2}=259.81\times 10^{7}\;{\mathrm{Pa}}^{2}$ for Equation (31) are obtained through trial and error. All models are run with a time step of 1ms and provide sensor data to the parameter estimation algorithm at 1000 Hz.

### 4.1. Estimating the Characteristic Curve of the Valve

**Figure 3:**
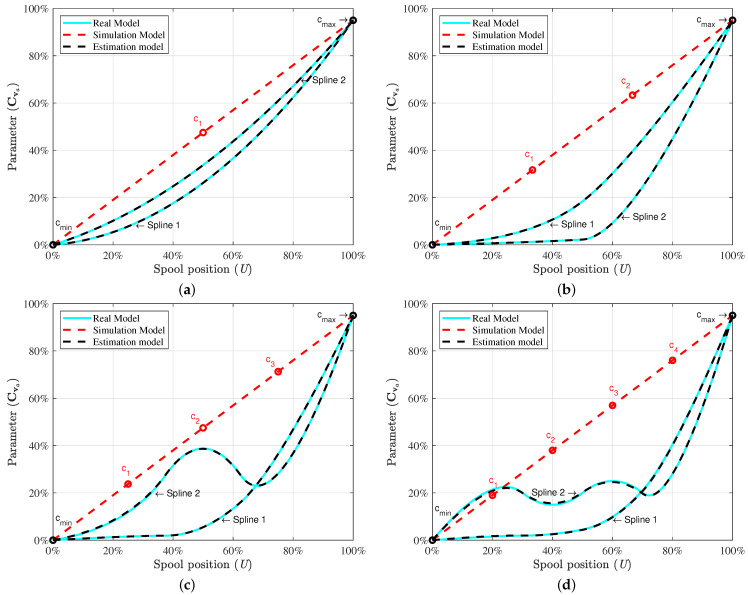
The estimation of the characteristic curves of the directional control valve by using the ADEKF with third-order B-spline interpolation. (**a**) Three-point B-spline estimation. (**b**) Four-point B-spline estimation. (**c**) Five-point B-spline estimation. (**d**) Six-point B-spline estimation.

In the real model, as only the minimum point $c_{\min}$ and the maximum point $c_{\max}$ on the characteristic curves are known at the *a*, *b*, *c*, and *d* ports of the directional control valve, the characteristic curves are generally unclear in the working cycles of the real model. The characteristic curves may vary from one valve to another and can be highly non-linear in the working cycle. In Figure 3, *Spline* 1 and *Spline* 2, which are in the cyan colour, are used to demonstrate the non-linear behaviour of the directional control valve in the real model.

The proposed parameter estimation algorithm can be used to estimate the characteristic curves of the real model with this limited information. To this end, the semi-empiric flow rate coefficients $C_{v_{a}}$, $C_{v_{b}}$, $C_{v_{c}}$, and $C_{v_{d}}$ are defined with the data control points between $c_{\min}$ and $c_{\max}$ in Equation (37). Equation (37) is further used in terms of the control point vector N in Equations (20)–(28) to estimate the characteristic curves.

**Figure 4:**
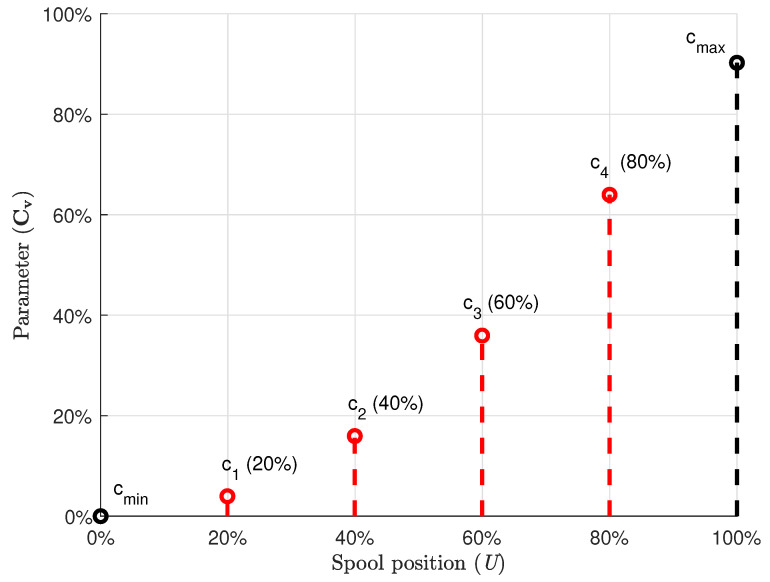
Data control points between $c_{\min}$ and $c_{\max}$ on the characteristic curve of the directional control valve.

For instance, in the case of Figure 4, the control point vector $N_{a}$ at port *a* of the directional control valve can be defined in terms of $c_{1}$, $c_{2}$, $c_{3}$, and $c_{4}$ as

(39) $$N_{a}={\left[\begin{array}{cccccc} c_{\min} & c_{1} & c_{2} & c_{3} & c_{4} & c_{\max} \end{array}\right]}^{T}.$$

To estimate the characteristic curve, three, four, five, and six control points are used in the control point vector $N_{a}$. As an example, these data control points for *Spline* 1 in each case are presented in Table 3.

The abscissa of vector $N_{a}$ represents the spool position *U*, whereas the ordinate of vector $N_{a}$ indicates the semi-empiric flow rate coefficients $C_{v_{a}}$ at port *a* of the directional control valve. The results of the second-order B-spline are described in Appendix A. The results of the third-order B-spline demonstrate the characteristic curve of the directional control valve relatively better in a working cycle, as shown in Figure 3. As can be seen, *Spline* 1 and *Spline* 2 of the third-order B-spline are drawn in each data control point estimation case. The dashed red-coloured line indicates the characteristic curve of the simulation model. The dashed black-coloured line demonstrates the estimation model. In Figure 3a, three points, $c_{\min}$, $c_{1}$, and $c_{\max}$, are used to estimate *Spline*1 and *Spline*2 of the real model. The characteristic curve of the estimation model precisely follows the real model in the case of three points. Further, the percentages of the root mean square error (RMSE) are described in the Table 3 for *Spline* 1 and *Spline* 2 to verify the observations.

Figure 3b shows the estimation of the characteristic curve when using four points $c_{\min}$, $c_{1}$, $c_{2}$, and $c_{\max}$. As can be seen in Figure 3b, the semi-empiric flow rate coefficient $C_{v_{a}}$ for *Spline* 2 changes with small increments until 52% opening of the spool as compared to *Spline*1 in the real model. After this point, the parameter $C_{v_{a}}$ increases sharply towards the maximum point $c_{\max}$. The difference of the estimated curve from the real model’s curve is indistinguishable. The RMSEs of these curves are given in Table 3. The relatively complicated non-linear behaviours of the directional control valve can be estimated by using five control points and six control points. This can be seen in Figure 3c,d. By using the estimated characteristic curves, the working conditions of the directional control valve can be predicted.

### 4.2. Convergence of the Vector Data Control Points

**Figure 5:**
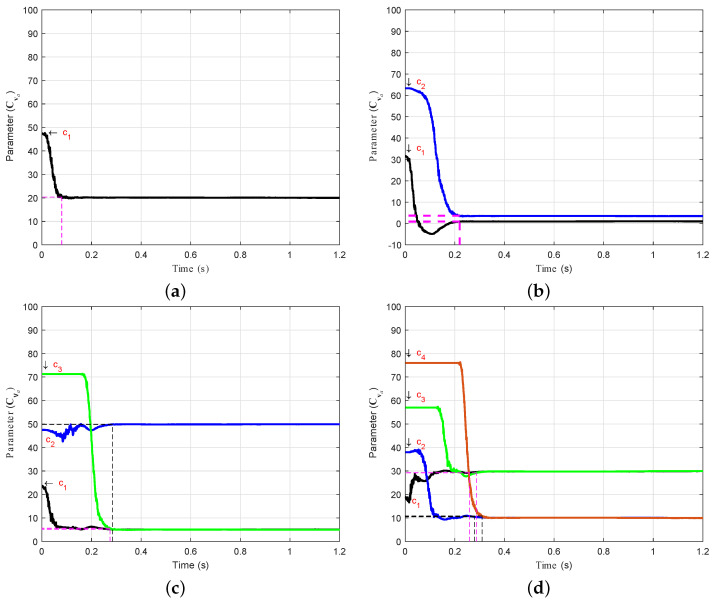
Convergence of the control points in the vector $C_{v_{a}}$ in the case of *Spline* 2. (**a**) Convergence of $c_{1}$ in the three-point estimation process. (**b**) Convergence of $c_{1}$ and $c_{2}$ in the four-point estimation process. (**c**) Convergence of $c_{1}$, $c_{2}$, and $c_{3}$ in the five-point estimation process. (**d**) Convergence of $c_{1}$, $c_{2}$, $c_{3}$, and $c_{4}$ in the six-point estimation process.

The convergence rate of the data control points in the parameter vector $C_{v_{a}}$ is further explained in Figure 5 to describe the estimation process. These plots demonstrate the convergence rate of data control points in the case of *Spline* 2, as presented in Figure 3. For instance (see Figure 5b), $c_{1}$ and $c_{2}$ converge towards the corresponding point on the curve of the real model at 0.22s. However, during the estimation process, $c_{1}$ briefly becomes negative, and shortly thereafter converges smoothly to the real model.

The curves of *Spline* 2 change into an S-shape during the working cycle, as shown in Figure 3c,d. In these cases, $c_{2}$, $c_{3}$, and $c_{4}$ converge at different simulation times according to the corresponding order in the vector $C_{v_{a}}$. Through the ADEKF algorithm, unknown curves start converging within a range of 0<t≤0.3s when using the three-, four-, five-, and six-point estimation techniques.

### 4.3. Accuracy Requirements of State Estimations

**Figure 6:**
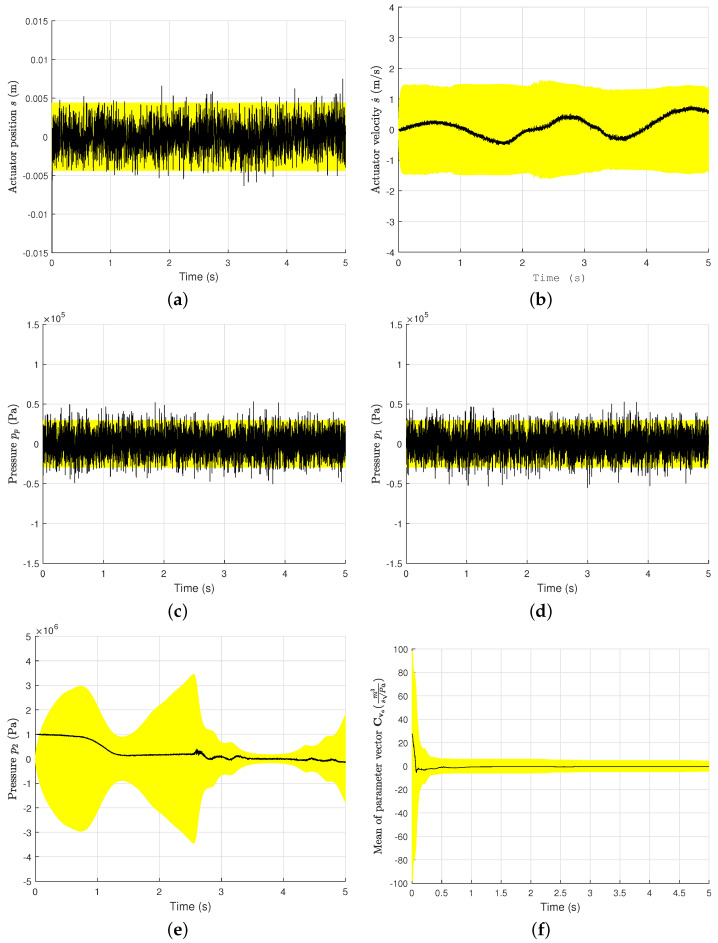
Requirements for the accuracy in the system states for parameter estimation. (**a**) Error in *s* with 95% CI. (**b**) Error in $\dot{s}$ with 95% CI. (**c**) Error in $p_{p}$ with 95% CI. (**d**) Error in $p_{1}$ with 95% CI. (**e**) Error in $p_{2}$ with 95% CI. (**f**) Error in parameter $C_{v}$ with 95% CI.

The successful application of the parameter estimation algorithm requires the accurate estimation of the system states x. To demonstrate this requirement, the errors in the estimated actuator position *s*, estimated actuator velocity $\dot{s}$, estimated pump pressure $p_{p}$, estimated piston side pressure $p_{1}$, estimated piston-rod side pressure $p_{2}$, and estimated parameter $C_{v_{a}}$ in the case of *Spline* 2 (described in Figure 3d) are shown in Figure 6. The errors in the estimated parameters $k_{p}$ and $k_{0}$ are presented in Appendix B. The average of the parameter vector $C_{v_{a}}$ at each time step is considered in Figure 6.

The errors are computed from ±1.96σ. Here, σ is the standard deviation calculated from the covariance matrix $P_{k}^{+}$ at each time step. These plots demonstrate the requirement of an accurate estimation of the system’s states to estimate the system’s parameters. As can be seen in Figure 6, the 95% confidence interval (CI) is used by the system states in the 5s simulation period. The errors in *s*, $\dot{s}$, $p_{p}$, $p_{1}$, and $\dot{s}$ fluctuate in the confidence interval. As indicated earlier, *s*, $\dot{s}$, and $p_{p}$ are measured in this example. The errors in the parameters $C_{v_{a}}$, $k_{p}$, and $k_{0}$ are also in the CI, as can be seen in the corresponding plots. The key to the parameter estimation is that the estimated system states should be in the 95% CI during the working cycle.

## 5. Conclusions

This work proposes the estimation of the parameters of a system by combining parameter estimation theories and curve-fitting methods. The ADEKF algorithm is introduced in the framework of a B-spline curve-fitting method. Using the proposed algorithm, the parameters can be defined as a vector containing a set of data control points. This algorithm is applied on a hydraulically driven four-bar mechanism to estimate the characteristic curves of a directional control valve. The double-step semi-recursive formulation and lumped fluid theories are used to model the four-bar mechanism and the hydraulic system, respectively. The measurements taken from the real system include the actuator position, pump pressure, and piston side pressure. The semi-empiric flow rate coefficient vector $C_{v_{a}}$ is defined with three to six data control points in order to define the characteristic curve of the directional control valve.

The unknown non-linear nature of the characteristic curves of the directional control valve are precisely estimated. The maximum RMSE observed in the estimation of the characteristic curves is 0.08%. This implies that the characteristic curves are accurately estimated. The data control points in the parameter vector $C_{v_{a}}$ converge in the range of 0<t≤0.3s in these estimation cases. To account for the system’s response, the estimation of the system’s state vector variables should be located in the 95% confidence interval. By using the estimated characteristic curves, important information about the discharge coefficient, pressure losses, and flow characteristics of the directional control valve can be interpreted. With this valuable information, manufacturers and users can monitor the condition of a system and make decisions about the repair and maintenance of hydraulically driven systems.

Applying the parameter estimation algorithm in the real world by using a multibody-based estimation model can enable the estimation of important parameters. This can be challenging, as the estimation model might not be as accurate as the real world necessitates. However, despite implementation challenges, the application of this parameter estimation algorithm will provide an interesting area for manufacturers and researchers. Manufacturers can use these parameters in condition monitoring, repair and maintenance, and the anticipation of product life cycles. With a product’s application history, important design changes can be introduced in future designs of the product. This will ultimately lead to more efficient MBS-based digital-twin applications through the use of real-time simulations and more sustainable future products.

## Figures and Tables

**Table 1:** Parameters of the hydraulic circuit.

Parameter	Symbol	Value
Pump flow rate	$Q_{p}$	0.001 $m^{3}/s$
Tank pressure	$p_{T}$	0.1 MPa
Volume of the hose *p*	$V_{p}$	3.42 $\times \;10^{-3}\;m^{3}$
Volume of the hose 1	$V_{h_{1}}$	3.42 $\times \;10^{-1}\;m^{3}$
Volume of the hose 2	$V_{h_{2}}$	3.42 $\times \;10^{-1}\;m^{3}$
Oil density	ρ	869 kg/m${}^{3}$
Hydraulic parameter	$k_{p}$	1600 MPa
Hydraulic parameter	$k_{0}$	0.5
Area of the piston	$A_{1}$	2 $\times \;10^{-3}\;m^{2}$
Area of the piston-rod	$A_{2}$	1.8 $\times \;10^{-3}\;m^{2}$
Length of the cylinder/piston	*l*	$\sqrt{3}$ m
Area of pressure relief valve	$A_{r}$	2.24 $\times \;10^{-12}m^{2}$
Area of directional control valve	$A_{d}$	1.96 $\times \;10^{-6}m^{2}$
Coulomb friction force	$F_{c}$	210 N
Static friction force	$F_{s}$	830 N
Stribeck velocity	$v_{s}$	1.25 $\times \;10^{-2}$ m/s
Coefficient of viscous friction	σ	330 Ns/m
Discharge coefficient	$C_{d}$	0.5
Area of throttle	$A_{R}$	2.24 $\times \;10^{-12}\;m^{2}$

**Table 2:** Properties of the real model, the estimation model, and the simulation model. Errors in the simulation model and the estimation model are given in comparison to the real model. $s_{1_{0}},\;p_{p_{0}}$, and $p_{1_{0}}$ represent the initial actuator position, the initial pump pressure, and the initial pressure on the piston side as the system states. The system parameters $C_{v_{a}}$, $C_{v_{b}}$, $C_{v_{c}}$, $C_{v_{d}}$, $k_{0}$, and $k_{p}$ represent the semi-empiric flow rate coefficient at the *a*, *b*, *c*, and *d* ports of the directional control valve, the flow gain, and the pressure flow coefficients, respectively.

Errors	Symbol	Real Model	Estimation Model	Simulation Model
State	$s_{1_{0}}$	$\sqrt{3}\;m$	1.62m	1.62m
State	$p_{p_{0}}$	7.6 MPa	5.6 MPa	5.6 MPa
State	$p_{1_{0}}$	1 MPa	2 MPa	2 MPa
Parameter	$C_{v_{a}}$	Non-linear	Linear	Linear
Parameter	$C_{v_{b}}$	Non-linear	Linear	Linear
Parameter	$C_{v_{c}}$	Non-linear	Linear	Linear
Parameter	$C_{v_{d}}$	Non-linear	Linear	Linear
Parameter	$k_{0}$	0.5	0.4	0.4
Parameter	$k_{p}$	1600 MPa	1500 MPa	1500 MPa

**Table 3:** Root mean square error in the estimation of the characteristic curve. The third and fourth columns represent the root mean square errors in *Spline* 1 and *Spline* 2, respectively.

Control Points	Control Point Vector $N_{a}$	RMSE	RMSE
Three points	$\left[\begin{array}{ccc} 0 & 5 & 10\\ 0 & 47.5 & 95 \end{array}\right]$	0.04%	0.03%
Four points	$\left[\begin{array}{cccc} 0 & 3.3 & 6.6 & 10\\ 0 & 31.6 & 63.3 & 95 \end{array}\right]$	0.05%	0.01%
Five points	$\left[\begin{array}{ccccc} 0 & 2.5 & 5 & 7.5 & 10\\ 0 & 23.7 & 47.5 & 71.2 & 95 \end{array}\right]$	0.06%	0.07%
Six points	$\left[\begin{array}{cccccc} 0 & 2 & 4 & 6 & 8 & 10\\ 0 & 19 & 38 & 57 & 76 & 95 \end{array}\right]$	0.07%	0.08%

**Figure A1:**
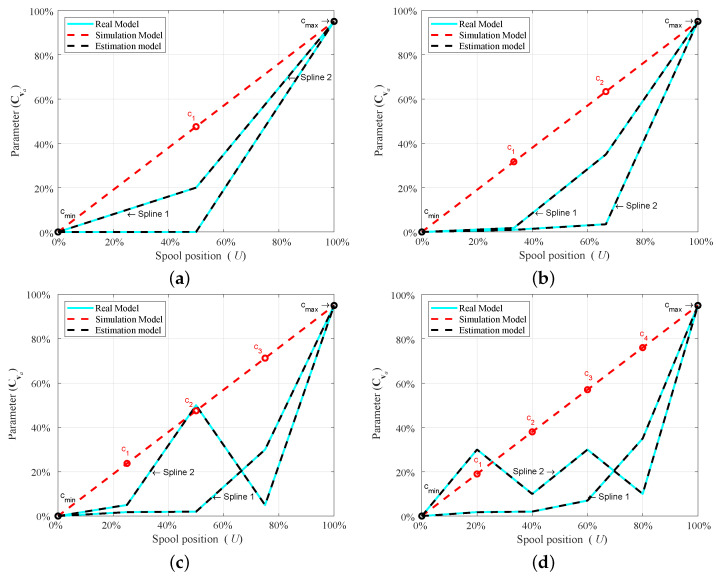
The estimation of the characteristic curve of the directional control valve by using the ADEKF algorithm with second-order (linear) B-spline interpolation. (**a**) Three-point B-spline estimation. (**b**) Four-point B-spline estimation. (**c**) Five-point B-spline estimation. (**d**) Six-point B-spline estimation.

**Figure A2:**
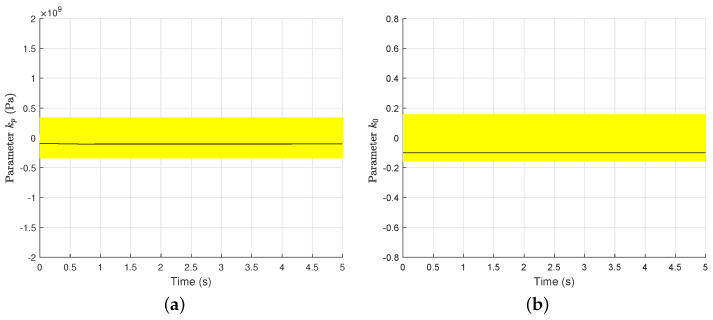
Requirements for the accuracy in the system states for parameter estimation. (**a**) Error in $k_{p}$ with 95% CI. (**b**) Error in $k_{0}$ with 95% CI.

## Data Availability

Not applicable.

## Appendix A. The Estimation of the Curve Using Second-Order (Linear) B-Spline Interpolation

When using second-order (linear) B-spline interpolation, the characteristic curves of the directional control valve are not continuous, as shown in the Figure A1. Therefore, to demonstrate the real-world application, it is recommended to use the third-order B-spline or above.

## Appendix B. Estimation of the Pressure Flow Coefficient and the Flow Gain

The estimation of the pressure flow coefficient $k_{p}$ and the flow gain $k_{0}$ in the case example is represented below.
